# Diagnosis, Follow-Up and Treatment Results in Thyroid Ophthalmopathy

**DOI:** 10.4274/tjo.93609

**Published:** 2015-08-05

**Authors:** Esra Savku, Kaan Gündüz

**Affiliations:** 1 Ankara University Faculty of Medicine, Department of Ophthalmology, Ankara, Turkey

**Keywords:** Clinical activity score, orbital decompression, orbital radiotherapy, Smoking, thyroid-associated ophthalmopathy

## Abstract

**Objectives:**

To discuss our follow-up and treatment results in thyroid-associated ophthalmopathy (TAO).

**Ma­te­ri­als and Met­hods:**

The records of 168 TAO cases who were followed at our clinic between October 1998 and October 2013 were reviewed retrospectively. The severity and activity of the disease were evaluated according to the criteria of the European Group on Graves’ Ophthalmopathy (EUGOGO) and Clinical Activity Score (CAS).

**Re­sults:**

Sixty-three men and 105 women participated in the study. The mean age of the patients was 42.3±12.4 years. Smoking habit was noted in 54.2% of the cases. Graves’ disease was the most common (80.4%) thyroid pathology accompanying TAO. TAO was mild in 64.4%, moderate-to-severe in 33.6% and severe in 2% of the eyes. Male gender was found as an independent risk factor for severity of the disease (p=0.040). TAO was in the active phase in 32.6% of the eyes. Older age and high thyroid receptor antibody titer were correlated with disease activity (P=0.031 and P<0.001, respectively). Thirty-four patients (20%) were treated for ocular findings. The most common treatment was systemic steroid therapy (12%); others included orbital decompression (5%), orbital radiotherapy (2%), and topical application of guanethidine (1%).

**Conclusion:**

Non-infiltrative phase and mild ocular findings were generally seen in TAO. Therefore, treatment is not recommended for many cases. Systemic steroid therapy is the most commonly used treatment modality in the active phase. However, orbital decompression surgery is necessary in a small number of cases with sight-threatening ocular findings.

## INTRODUCTION

Thyroid-associated ophthalmopathy (TAO), also known as Graves’ ophthalmopathy, infiltrative ophthalmopathy or thyroid eye disease, is the most important non-thyroidal symptom of Graves’ disease (GD). Ocular involvement frequently accompanies thyroid dysfunction, although clinical ophthalmopathy may also arise in euthyroid patients.^1^

TAO is an autoimmune disease resulting from the antibody response to an antigen common to the thyroid and retroorbital tissue. This antigen seems to be primarily located in the fibroblasts of retroorbital tissue. This theory is supported by findings of thyroid stimulating hormone receptor (TSH-R) mRNA expression in orbital fibroblasts^2,3^ and elevated thyroid receptor antibody (TRAb) titers in patients with severe ophthalmopathy.^4,5^

Although ocular symptoms occur frequently in autoimmune thyroid disease, the majority of patients have mild to moderate clinical ophthalmopathy. More rarely, patients develop optic neuropathy or corneal perforation leading to severe ophthalmopathy and subsequent vision loss. Diagnosis is usually made on the basis of clinical findings; thyroid function tests, thyroid antibodies and orbital imaging methods also assist diagnosis.

There is currently no treatment to prevent the development of ophthalmopathy. Symptomatic approaches are sufficient to treat the disease in mild cases. However, moderate to severe cases of TAO may require multiple medical and/or surgical interventions. In these cases, close monitoring and the proper sequencing of treatment are extremely important. Medical treatment should be initiated as early as possible in the active phase of the disease. Rehabilitative surgical procedures are indicated for the chronic phase and should follow a certain sequence: decompression surgery is first, followed by strabismus correction, and finally eyelid surgery.

The course of thyroid ophthalmopathy is also affected by thyroid gland oriented treatment choices;^6,7^ therefore, close follow-up of these patients via the cooperation of endocrinologists and ophthalmologists is necessary.

In this study, our aim was to evaluate demographic characteristics, smoking habits, comorbid diseases and the outcomes of treatment modalities against the thyroid and eye diseases in patients diagnosed with TAO and referred from endocrinology and other ophthalmology clinics to our clinic as a tertiary referral center.

## MATERIALS AND METHODS

The study included 306 eyes of 168 TAO patients who were seen and followed for at least 6 months in the tumor unit of Ophthalmology Department at Ankara University Faculty of Medicine between September 1998 and September 2013. The study was organized and conducted in accordance with the principles of the Helsinki Declaration. The patients signed an informed consent form before proceeding with any examinations or treatments. The study was approved by the local ethics committee of Ankara University.

The medical reports of all the patients were analyzed retrospectively. Age, gender, duration of thyroid disease and ocular involvement, subtypes of thyroid disease, accompanying diseases, cigarette use, family history, previous treatment modalities against thyroid disease, ocular findings and ocular treatments administered in our clinic were recorded.

During the follow-up, all patients underwent best corrected visual acuity (BCVA) measurement using the Snellen chart, slit-lamp examination of the anterior segment, intraocular pressure measurement using Goldmann aplanation tonometry, detailed fundus examination and automated perimetry (AP). Proptosis was assessed by Hertel exophthalmometry and any measurement higher than 21 mm was accepted as pathological. Orbital magnetic resonance imaging (MRI) or computed tomography (CT) was performed when necessary.

The severity and activity of ocular involvement were evaluated according to the European Group on Graves Ophthalmopathy^8,9^ (EUGOGO) and Clinical Activity Score9 (CAS) criteria, respectively. These classifications are shown in Tables 1 and 2.

Mild cases were seen at 6-month intervals; other cases were followed-up more frequently. All the patients were given preservative-free artificial tears. The patients with eyelid retraction were treated with 5% guanetidine sulfate drops and the patients with glaucoma were treated with topical anti-glaucoma medication. The patients with moderate to severe, active ocular involvement received oral or intravenous steroids for 12 weeks. Oral steroid treatment consisted of methylprednisolone at a daily starting dose of 80 mg/d which was decreased in 8 mg decrements each week to 4 mg/d in week 11, followed by 4 mg on alternate days in week 12. Intravenous steroid treatment consisted of methylprednisolone at 500 mg per week for the first 6 weeks and 250 mg per week for the following 6 weeks. Patients with sight-threatening TAO were treated with pulse steroid therapy (1000 mg/d methylprednisolone for 3 days) followed by urgent orbital decompression. Orbital radiotherapy (OR) was chosen for patients with diplopia due to extraocular muscle (EOM) involvement; patients received a total of 20 Gy external radiotherapy (ERT) in divided doses.

For statistical analysis, Snellen visual acuity was converted to logMAR. Following univariate analysis, multivariate logistic regression analysis was performed as necessary. Level of significance was accepted as α=0.05.

## RESULTS

The study included 306 eyes of 168 patients. Mean age of the patients was 42.3±12.4 (range, 18-82) years. There were 105 (62.5%) women and 63 (37.5%) men. There was no statistical difference in age distribution between the male and female patients (p=0.837). Just over half (54.2%) of the patients had a history of cigarette use; their mean daily cigarette use was 10 cigarettes (range, 2-80) and mean duration of cigarette use was 20 years (range, 3-40 years). One quarter (25.1%) of the patients had a close relative with thyroid disease. Regarding other diseases, 14.9% were hypertensive, 10.2% were diabetic, and 2.3% had a comorbid autoimmune disease (myasthenia gravis in 2 patients, multiple sclerosis in 1 patient, and vitiligo in 1 patient).

At time of presentation, euthyroidism was the most common thyroid status (64.9%), followed by hyperthyroidism (27.4%) and hypothyroidism (7.7%). Among patients with thyroid disease, 135 patients (80.4%) had Graves’ disease, while the remaining 33 patients had other thyroid diseases (Hashimoto’s thyroiditis in 8.3%, multinodular goiter in 8.3% and thyroid cancer in 3%). Case histories acquired from patients and discharge reports revealed that the most common thyroid treatment administered in various centers was anti-thyroid medication (68.5%); other treatments included thyroidectomy (14.3%) and radioactive iodine therapy (10.7%).

Mean duration of ocular involvement was 12 months (range, 1-300 months). The majority (82.1%) of patients had bilateral involvement. Median visual acuity was 0.0 logMAR (range, 0.0-1.8 logMAR). Mean intraocular pressure was 16.5±2.9 mmHg (range, 10-32 mmHg) and glaucoma was detected in 21 eyes (6.3%). Ocular findings included eyelid retraction in 68%, upper eyelid lag on down gaze in 54.8%, lagophthalmos in 6.9% and ptosis in 2% of the eyes. Mean Hertel exophthalmometry value was 20.3±3.2 mm (range, 14-30 mm), and proptosis was determined in 47% of the eyes. Compressive optic neuropathy was detected at a rate of 2%, while serious corneal involvement was not present in any case.

The most common form of diplopia was inconstant diplopia (8.3%), followed by intermittent diplopia in 6.5% and constant diplopia in 2.4% of the patients. On examination, limited eye movement was observed in 32.4% of the eyes, although only 12 cases (7.1%) exhibited manifest deviation. Orbital CT or MRI in 214 eyes of 119 patients revealed proptosis with EOM involvement in 63.6%, proptosis alone in 20.8%, and EOM involvement alone in 2.7%. The most commonly affected muscle was the inferior rectus muscle (89.6%), followed by the medial rectus (69.6%), lateral rectus (17.9%) and superior rectus muscles (15.1%).

Evaluation of disease severity indicated mild involvement in 197 eyes (64.4%), moderate-to-severe involvement in 103 eyes (33.6%) and severe involvement in 6 eyes (2%) (Table 3). Due to the small number of eyes with severe involvement, these cases were combined with moderate-to-severe cases for statistical comparison and evaluation of factors that influence disease severity. Univariate analysis revealed that TAO severity was associated with gender, thyroid function and thyroid treatment (Table 4). However, in multivariate logistic regression analysis, gender emerged as the unique independent risk factor for TAO severity (Table 5).

TAO disease was in active phase in 100 eyes (32.6%) and inactive phase in 206 eyes (67.4%) (Table 3). Mean CAS was 2.21±1.36. Eyelid edema (80.7%) and conjunctival hyperemia (52.6%) were the most common findings. Univariate analysis revealed that TAO activity was associated with age and TRAb level (Table 6), and multivariate logistic regression analysis confirmed these factors as independent risk factors for TAO activity (Table 7).

Thirty-four patients (20%) were treated for ocular findings. The most frequently used treatment was systemic steroids (12%), followed by orbital decompression (5%), orbital radiotherapy (2%) and topical guanetidine sulfate (1%).

Systemic steroids were administered to 18 patients orally and to 3 patients parenterally. Among these 21 patients, 13 patients completed the course of treatment and came to the 3rd month follow-up. TAO activity was decreased in 11 patients (84.6%) and remained unchanged in 2 patients (15.4%). Mean CAS was 4.19±1.16 at baseline and decreased to 2.15±1.12 after treatment. TAO severity lessened in 6 patients (46%) and remained unchanged in 7 patients (54%). Mean proptosis was 21.0 mm (15-28 mm) at baseline and decreased to 20.0 mm (13-26 mm) after treatment.

**Figure 1:**
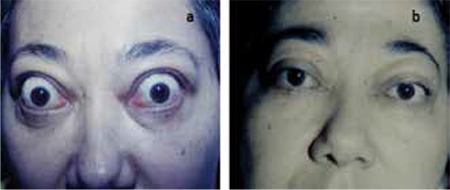
A patient before (a) and 3 months after (b) orbital radiotherapy

Three patients (4 eyes) with diplopia and EOM dysfunction were treated with 20 Gy ERT. In the 3rd month of treatment, improvement in both diplopia and eye movement restriction were reported (Figure 1). None of the patients developed radiation cataract or retinopathy during the follow-up period (mean duration, 6 months; range, 9-12 months).

**Figure 2:**
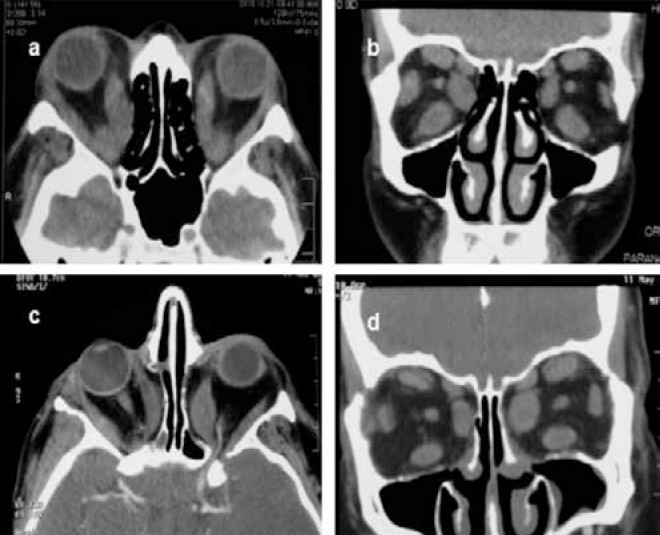
Orbital computed tomography images of a patient before (a, b) and after (c, d) decompression surgery. Proptosis was reduced in both eyes postoperatively

Eight of the patients (9 eyes) underwent medial and lateral (balanced) orbital decompression. Preoperative and postoperative proptosis values were 25 mm (23-30 mm) and 20 mm (19-25 mm), respectively (Figure 2). In patients with preoperative esotropia, angle of deviation increased by an average of 20 PD and diplopia worsened.

## DISCUSSION

The pathogenesis of TAO is not completely understood, though genetic and environmental factors are believed to play role in its development. In the first multi-center EUGOGO study, it was reported that 33% of the patients had a first-degree relative having thyroid disease.^8^ Similarly, in our study this rate was 39.2%.

Patients with Graves’ disease often present with ocular symptoms which can range along a spectrum from mild eyelid retraction and soft tissue involvement to dysthyroid optic neuropathy and corneal perforation.^8^ In the current study, the most common clinical finding was eyelid retraction, followed by proptosis. Similarly, Sasim et al.^10^ reported eyelid retraction (76%) and proptosis (58%) as the most common symptoms in their study. Lim et al.^11^ reported that proptosis was the most common (65.5%) clinical finding in their series, followed by eyelid retraction (53.4%) and EOM involvement (8.6%).

The combination of eyelid retraction and proptosis increases the likelihood of corneal exposure. Tightness of the inferior rectus muscle eliminates the protective Bell’s phenomenon (upward movement of the globe while closing the eye). The situation can be further worsened by reduced tear production due to lacrimal gland inflammation. Consequently, the incidence of dry eye in these patients is higher than in the general population. Patients may develop corneal involvement secondary to dry eye ranging from punctate epitheliopathy to corneal perforation. Therefore, local lubricants are recommended for protection of the cornea, with preference for preservative-free formulas to avoid inflammation due to preservatives. All the patients in our study were given topical artificial tears and gel and none of them developed serious corneal involvement.

Based on the disease severity classification, the majority of eyes in our study (64.4%) had mild involvement, and severe involvement was rare (2%). Similarly, Tanda et al.^12^ also observed that mild involvement was at a high rate (73.6%) and severe involvement was rare (5.3%). Sasim et al.^10^ also reported that symptoms were usually mild (64%), although the frequency of severe involvement was higher (9%). In a multi-center EUGOGO study conducted by Prummel et al.,^8^ they reported that the ocular findings were mild in 40%, moderate-to-severe in 32% and severe in 28% of the cases. The higher rate of severe disease in that study may be due to the fact that EUGOGO centers were referral centers and so more complicated cases were consulted.

In the current study, male gender was found as the unique independent risk factor for TAO severity. While 34.5% of mild TAO cases were men, this ratio increased to 49.5% in severe cases. In other words, the female to male ratio was 1.9:1 in mild cases versus 1:1 in severe cases. Kendler et al.^13^ also showed a positive correlation between disease severity and male gender. However, some studies reported that the male ratio in severe cases was higher than in mild cases, but this difference could be statistically insignificant.^12,14^

In humans, exposure to certain environmental xenobiotics, including those in cigarettes, leads to loss of tolerance to self proteins in individuals with genetic predisposition.^15^ This explains why cigarette use is an important risk factor for the development of Graves’ disease and ophthalmopathy. Various studies have reported that cigarette use increases the incidence of ophthalmopathy 2-10 times.^10,14,16,17,18^ Other studies have also shown that smoking habit relates to EOM volume rise,^19^ optic neuropathy development20 and TAO severity.^14,20^ In the current study, we also showed a higher rate of cigarette use in severe TAO cases, but this result was not statistically significant.

Currently, TAO is considered to be an autoimmune process; the immune system recognizes a common antigen of the thyroid and orbital tissues. Orbital fibroblasts seem to be the primary location of this antigen. There is evidence in the literature indicating that TSH-R mRNA is expressed in orbital fibroblasts.^2,3^ If orbital TSH-R is actually responsible for ophthalmopathy, an association is expected between TRAb titers and clinical findings of TAO. Many studies have reported the relationship between TRAb levels and TAO severity and/or activity.^4,5^ However, when an immune attack ends, ocular findings do not resolve completely despite lower TRAb levels. For this reason, some researchers believe that TRAb level is not associated with TAO severity, but indicates activity.^21^ Similarly, we found higher TRAb levels in patients with active TAO.

Eighty percent of our patients were observed without any treatment. Approximately 65% of the patients had mild TAO symptoms; the remaining 15% refused treatment or could not be treated because of their comorbid diseases.

Systemic steroids are the first-line treatment for patients with active TAO. They inhibit cytokine release, thereby reducing the synthesis of glycoaminoglycan (GAG) in orbital fibroblasts. In a study by Kauppinen-Mäkelin et al.^22, 33^ TAO patients were treated with intravenous or oral steroids and TAO activity decreased in the 3rd and 12th months of treatment as compared to before treatment. Kahaly et al.^23^ reported a decrease in TAO severity and activity in the 3rd month in 18 of 35 (51%) patients treated with oral steroids and 27 of 35 (77%) patients treated with intravenous steroids. Aktaran et al.^24^ also mentioned a decrease in TAO severity and activity in the 3^rd^ month in 13 of 27 (48%) patients treated with oral steroids and 18 of 25 patients treated with intravenous steroids. In our study, 21 patients were treated with intravenous or oral steroids and 13 of them came for follow-up. Improvement was seen in disease severity in 46% and in disease activity in 84.6% of these patients. Because oral steroids were preferred in the majority of our cases, the ratio of our cases with decreased disease severity was similar to the results of the oral steroid group in the mentioned study (46% vs. 51%).

Another treatment option for thyroid ophthalmopathy is radiotherapy. Orbital radiotherapy (OR) is preferred due to high radiosensitivity of lymphocytes infiltrating the globe, in addition to the general anti-inflammatory effects of radiotherapy.^25^ OR also suppresses the production of GAG in the orbital fibroblasts.^26^ This treatment is mainly effective on impaired eye movements. Mourits et al.^27^ treated 30 patients with 20 Gy ERT and reported recovery of restricted EOM movement and diplopia in 18 patients (60%) in the 6^th^ month of treatment. Similarly, Prummel et al.^28^ treated 44 patients with 20 Gy ERT and reported improvement in EOM movement and reduced diplopia in 23 patients (57.5%) in the 12^th^ month of treatment. Both studies demonstrated a significant difference in response to treatment between the treatment and control groups. However, Gorman et al.^29^ treated one of the eyes of 21 patients with 20 Gy ERT; they observed only slight improvement in ocular findings in the treated eye and detected no statistically significant difference in response to treatment between the treated and untreated eyes of patients in the 6^th^ month of treatment. In our study, 3 patients were treated with 20 Gy ERT and showed marked improvement in EOM movement and diplopia in the 6^th^ month of treatment. OR is generally well tolerated and safe, although it increases the incidence of dry eye due to lacrimal gland damage. Cataract is also a possible complication, but this complication can be minimized by low-dose radiotherapy. Radiation retinopathy is extremely rare. In the current study, the total dose of 20 Gy was administered in divided doses in 10 days of 2 weeks; none of our patients developed radiation cataract or retinopathy.

When response to medical treatment is limited or absent, urgent surgical intervention is necessary in sight-threatening TAO cases. Other surgical procedures are performed in chronic phase when fibrosis takes place of inflammation and systemic steroids or radiotherapy are no longer effective. If orbital decompression is indicated, it must be performed before other procedures. The most common decompression procedure is medial-lateral wall (balanced) decompression. Shepard et al.^30^ performed balanced decompression in 18 eyes of 11 patients and reported a mean reduction in proptosis of 4.6 mm. Similarly, using the same procedure Graham et al.^31^ achieved a mean reduction in proptosis of 4.1 mm in 63 eyes of 40 patients and Sellari-Franceschini et al.^32^ reported a mean reduction of 5.3 mm in 140 patients. In the current study, balanced decompression was performed in 9 eyes of 8 patients, with a postoperative mean reduction in proptosis of 5.0 mm, consistent with the literature.

Diplopia is one of the most important side effects of decompression surgery. Postoperative diplopia develops more frequently in patients with preoperative EOM dysfunction than in patients with normal ocular motility.^33^ Shepard et al.^30^ and Graham et al.^31^ reported postoperative diplopia in 2 of 11 cases and 4 of 40 cases, respectively. Sellari-Franceschini et al.^32^ reported new-onset or worsened diplopia in 20% of their patients. In the current study, angle of deviation increased and diplopia worsened postoperatively in our two patients having preoperative esotropia and diplopia.

Correcting diplopia is more challenging in patients who have undergone decompression surgery. The procedure widens the orbital space, which leads to changes in EOM paths. Diplopia may be treated temporarily by closing one eye or using prisms. However, strabismus surgery is the main treatment in these cases. Strabismus surgery is based on the recession of fibrotic ocular muscles. Following decompression surgery, strabismus surgery was recommended to our two patients whose diplopia worsened, but they refused the procedure.

The symptoms of TAO are usually mild; therefore, monitoring is recommended in the majority of cases. The most commonly used treatment in the active phase is systemic steroids. In a few patients with severe, sight-threatening ocular involvement, decompression surgery is necessary.

## Figures and Tables

**Table 1:**
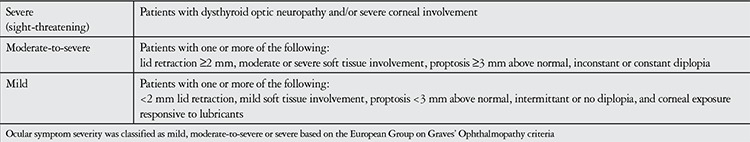
European Group on Graves’ Ophthalmopathy (EUGOGO) severity classifications^8^

**Table 2:**
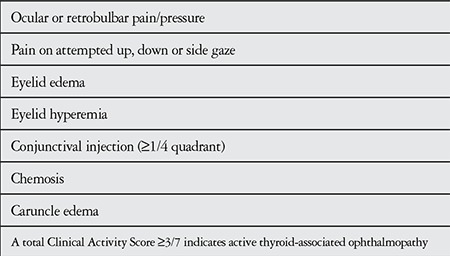
Clinical Activity Score (CAS)^9^

**Table 3:**
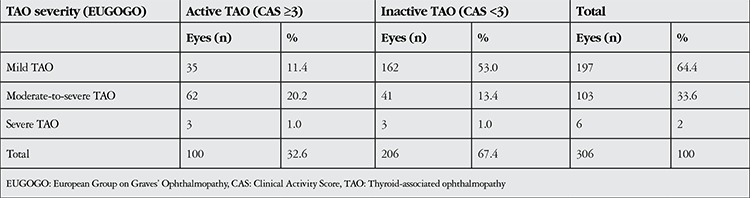
Distribution of patients by ocular symptom severity and activity

**Table 4:**
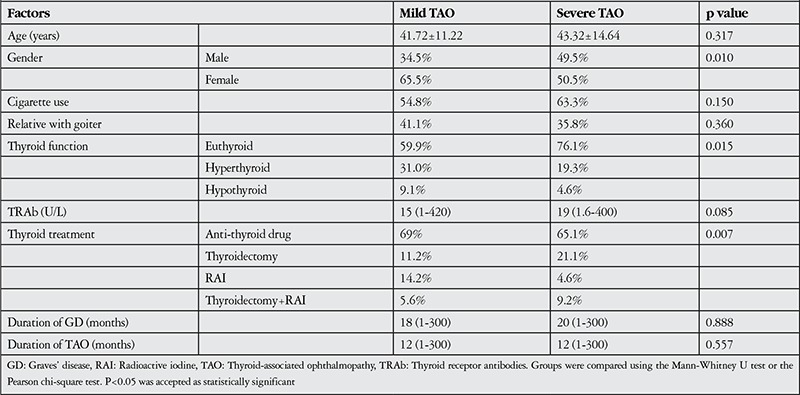
Factors affecting thyroid-associated ophthalmopathy severity (univariate analysis)

**Table 5:**
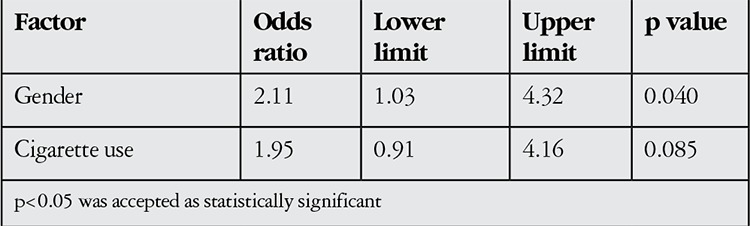
Factors affecting thyroid-associated ophthalmopathy severity (multivariate logistic regression analysis)

**Table 6:**
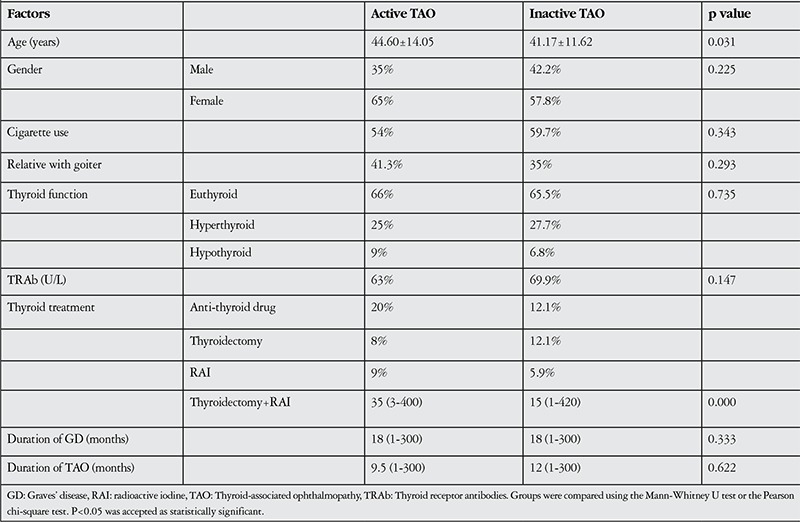
Factors affecting thyroid-associated ophthalmopathy activity (univariate analysis)

**Table 7:**
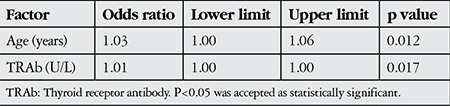
Factors affecting thyroid-associated ophthalmopathy activity (multivariate logistic regression analysis)
